# Diversifying selection and host adaptation in two endosymbiont genomes

**DOI:** 10.1186/1471-2148-7-68

**Published:** 2007-04-30

**Authors:** Jeremy C Brownlie, Marcin Adamski, Barton Slatko, Elizabeth A McGraw

**Affiliations:** 1 School of Integrative Biology, University of Queensland, Brisbane, QLD 4072 Australia; 2 Molecular Parasitology Division, New England Biolabs, Ipswich, MA 01938 USA

## Abstract

**Background:**

The endosymbiont *Wolbachia pipientis *infects a broad range of arthropod and filarial nematode hosts. These diverse associations form an attractive model for understanding host:symbiont coevolution. *Wolbachia*'s ubiquity and ability to dramatically alter host reproductive biology also form the foundation of research strategies aimed at controlling insect pests and vector-borne disease. The *Wolbachia *strains that infect nematodes are phylogenetically distinct, strictly vertically transmitted, and required by their hosts for growth and reproduction. Insects in contrast form more fluid associations with *Wolbachia*. In these taxa, host populations are most often polymorphic for infection, horizontal transmission occurs between distantly related hosts, and direct fitness effects on hosts are mild. Despite extensive interest in the *Wolbachia *system for many years, relatively little is known about the molecular mechanisms that mediate its varied interactions with different hosts. We have compared the genomes of the *Wolbachia *that infect *Drosophila melanogaster*, *w*Mel and the nematode *Brugia malayi*, *w*Bm to that of an outgroup *Anaplasma marginale *to identify genes that have experienced diversifying selection in the *Wolbachia *lineages. The goal of the study was to identify likely molecular mechanisms of the symbiosis and to understand the nature of the diverse association across different hosts.

**Results:**

The prevalence of selection was far greater in *w*Mel than *w*Bm. Genes contributing to DNA metabolism, cofactor biosynthesis, and secretion were positively selected in both lineages. In *w*Mel there was a greater emphasis on DNA repair, cell division, protein stability, and cell envelope synthesis.

**Conclusion:**

Secretion pathways and outer surface protein encoding genes are highly affected by selection in keeping with host:parasite theory. If evidence of selection on various cofactor molecules reflects possible provisioning, then both insect as well as nematode *Wolbachia *may be providing substances to hosts. Selection on cell envelope synthesis, DNA replication and repair machinery, heat shock, and two component switching suggest strategies insect *Wolbachia *may employ to cope with diverse host and intra-host environments.

## Background

Intracellular bacterial symbiont associations are extremely common in invertebrates. The capacity for these symbionts to shape host biology is immense and includes documented effects on host reproduction [1], food preference [2], locomotion [3], and interspecific competition [4]. Teasing apart the contributions of insect and symbiont genomes to such multi-organism determined phenotypes is necessary if the evolution and ecology of both partners are to be understood. This can be challenging, because the complex biotic interaction also makes these systems less tractable experimentally. Comparative study of sequenced symbiont genomes and their relatives is offering new means to direct empirical study of symbiosis [5].

The endosymbiont *Wolbachia pipientis *infects a wide range of arthropod and filarial nematode hosts. Across its host range the microbe is associated with diverse phenotypic outcomes. The *Wolbachia*-nematode associations are mutualistic while all other associations could be described as commensal or parasitic in nature. In nematodes the infection is confined to the nematode reproductive tract and the hypodermal tissue where the microbe plays an integral role in host viability and reproduction [6,7]. Phylogenies of *Wolbachia *and their nematode hosts are congruent, reflecting a long history of strict vertical transmission [8]. Tight associations like these are predicted to generate genome reduction [9], as host support of symbiont requirements leads to degradation and loss of the genes in these redundant pathways. Consistent with this prediction, the genome of the *Wolbachia *strain that infects *Brugia malayi *(*w*Bm) is much smaller and highly streamlined relative to the genomes of free-living bacteria and other *Wolbachia *[10,11].

The *Wolbachia*-arthropod association, in contrast, is more fluid in nature. Infections are not fixed in populations and most appear to be mild in their effects on host fitness [12,13]. Horizontal transmission among host lineages is common on a phylogenetic time scale, meaning closely related *Wolbachia *can be found in taxonomically diverse hosts [14]. Infections can be found in numerous somatic tissues as well as the gonads [15]. The presence of *Wolbachia *in insect hemolymph in combination with recent experimental work also suggests that the bacteria may be exposed to extracellular environments for sustained periods [16]. Across the arthropods *Wolbachia *also induces a broad range of reproductive manipulations including feminization, male killing, cytoplasmic incompatibility, and parthenogenesis [1,17,18]. The pattern of *Wolbachia *tissue distribution, infection densities, induced fitness effects, and reproductive manipulations vary greatly within the arthropods and are the result of host and bacterial genotype interactions [19-22].

Here we report the results of genome wide screens for the presence of diversifying selection in the *Wolbachia *that infect the filarial nematode, *Brugia malayi w*Bm [10] and the insect *Drosophila melanogaster*, *w*Mel [11]. Per gene estimates of nonsynonyous substitution per nonsynonymous site versus synonymous substitution per synonymous site (*d*_*N*_/*d*_*S*_) in the *Wolbachia *relative to the outgroup species, *Anaplasma marginale *[23] were used to infer past history of positive selection [24]. This approach has been utilized previously to explore the genetic basis of complex phenotypes in a diverse range of taxa [24-29]. By identifying key molecular adaptations in each of the two *Wolbachia *lineages, we sought to shed light the mechanistic basis of the *Wolbachia *symbiosis and how it might vary with respect to different hosts. We hypothesized that genes whose encoded proteins were involved with secretion or were localized to the *Wolbachia *cell surface would show evidence of strong selection due to their interaction with the host. We also expected to find evidence of selection on pathways that could be used for host provisioning in *w*Bm. The screen confirmed both these hypotheses. The genomic comparisons also revealed possible points of host provisioning in *w*Mel and strategies *Wolbachia *may have evolved for coping with diverse hosts and intra host environments.

## Results & Discussion

### Summary

**Figure 1:**
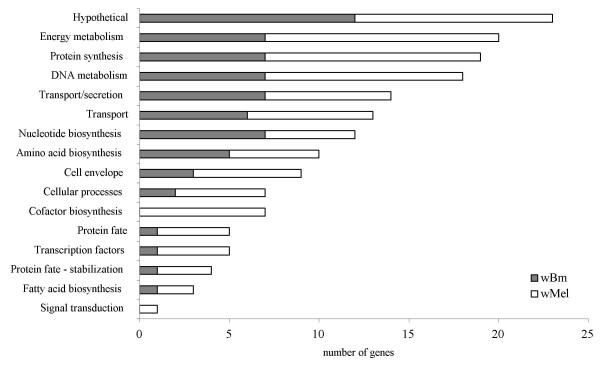
**Positive selection by functional role**. Total number of genes exhibiting significant positive selection by functional role in the *w*Bm and *w*Mel genomes.

Of the 591 loci examined, 60 in *w*Bm and 101 in *w*Mel bore signatures of positive selection (see Additional file 1). The proportion of genes affected by diversifying selection in *Wolbachia *was higher than reports from other screens in bacteria [26] and may reflect the well-documented phenomenon of rapid evolution in endosymbionts [30-32]. The small effective population sizes of these bacteria would predict more rapid fixation of nonsynonymous mutations due to drift and hence generate higher average ratios of d_N _to d_S _[33]. The distribution of significant genes was not clumped with respect to genome position (data not shown) with the exception of the ribosomal protein encoding genes, which are members of an operon. Excluding hypothetical and unknown groups, the significant genes represent 13  functional classes (GenomeAtlas annotation) in *w*Bm and 15 in *w*Mel (TIGR annotation) (Fig. 1 and see Additional file 1). Genes comprising the biological role categories nucleotide biosynthesis, amino acid biosynthesis, and transport/secretion were similarly affected by diversifying selection in both genomes. In general, evidence of selection was more common in the *w*Mel genome. Larger numbers of genes in the role categories of DNA metabolism, energy metabolism, protein synthesis, cell envelope synthesis, cofactor biosynthesis, and protein fate were disproportionately affected in *w*Mel (Fig. 1). We have reviewed the gene composition for several of these functional categories and speculate on their role in the evolution of insect vs. nematodee symbiont associations.

### Purifying selection

An examination of the *d*_*N*_*/d*_*S *_ratios also highlighted those genes experiencing extreme levels purifying selection in either of the *Wolbachia *lineages. A total of 323 genes in the *w*Bm lineage and 250 genes in *w*Mel had a ratio < 1.0. We then examined only the most severely affected genes (*d*_*N*_*/d*_*S *_< 0.2) in each lineage and asked whether genes from any of the functional categories were over-represented. Most of the major functional classes were represented by only a small number of genes. The exceptions were the categories of synthesis and modification of ribosomal proteins in both genomes and the biosynthesis and degradation of cell envelope in *w*Bm only. The former represented ~15% of genes with *d*_*N*_*/d*_*S *_< 0.2 and the latter 8% of the genes for *w*Bm. The extreme conservation in ribosomal protein evolution is not surprising given their essential and conserved cellular functions for all kingdoms of life. Purifying selection on cell envelope component genes in *w*Bm is interesting given that these same genes are experiencing diversifying selection in *w*Mel (see Additional file 1). The *Wolbachia *cell envelope may be exposed to vastly different environments in the insect versus nematode hosts. Differences in how selection is operating on the genes encoding membrane proteins may reflect adaptation to lineage specific ecological niches (see *Direct contact with the host *below).

### Evidence of provisioning

**Figure 2:**
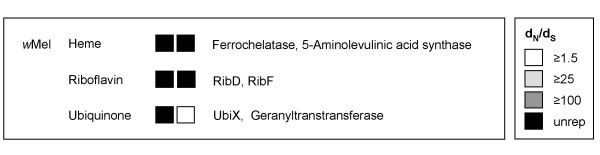
**Cofactor biosynthesis**. Specific genes and d_N_/d_S _exhibiting selection (BH p = 0.001 model and Fisher's exact test) by select functional role for each genome. Each box corresponds to a significant gene in the sub role and degree of shading indicates magnitude of d_N_/d_S_. "Unrep" indicates the ratio was at the reportable limit of codeml (see methods). Names of gene products are listed in order to the right of boxes. See Additional file 1 for individual *p *values, gene ids/descriptions, WD#s, and d_N _& d_S _values.

The basis of *Wolbachia*'s dependence on its host and the nature of any benefits provided to hosts are two fundamental unknown aspects of this symbiotic association. The completed genome sequences of *w*Bm and *w*Mel [10,11] have only recently advanced our understanding of what *Wolbachia *can and cannot synthesize and what it may be transporting across its membrane. Symbiont provisioning of insect hosts is hypothesized for many associations and has been documented in numerous insects including; aphids [34], tsetse flies [35], rice weevils [36], and ants [37]. Evidence of provisioning would not be surprising in the nematode relationships as *Wolbachia *is clearly acting as a mutualist. Arthropod *Wolbachia *have traditionally been thought of as parasitic and therefore the presence of diversifying selection on a number of cofactor biosynthesis genes is particularly exciting (Fig. 2 & see Additional file 1).

Both *Wolbachia *genomes lack complete pathways for *de novo *synthesis of coenzyme A, NAD, biotin, lipoate, ubiquinone, and folate; presumably the host supplements these compounds [10,11]. Several genes that encode for components of these disrupted biosynthetic pathways show evidence of positive selection in *w*Mel and may reflect the molecular evolutionary process of integrating host and symbiont systems (Fig. 2 & see Additional file 1). Selection on genes in these same pathways was also detected in *w*Bm, but under less stringent rejection criteria (see Additional file 1). Unlike the above listed cofactors, riboflavin biosynthesis pathways are complete in both *Wolbachia *strains. Evidence for positive selection on riboflavin synthesis was present in *w*Mel (Fig. 2, Model p < 0.001, & Fisher's p < 0.001) and again in *w*Bm under slightly less stringent criteria (see Additional file 1). Symbiont provisioning of riboflavin has been documented in both weevil-SOPE and aphid-*Buchnera *associations [36,38]. Two members of the heme biosynthetic pathway (of seven genes in total) were affected by selection in *w*Mel. Additional genes in the heme biosynthesis pathway were also identified in both *w*Bm and *w*Mel when less stringent rejection criteria were applied (see Additional file 1). An examination of the *Brugia malayi *genome [10] suggests that the nematode may be incapable of synthesizing its own heme and therefore it is possible that *w*Bm *Wolbachia *may be provisioning its host with heme intermediates. Although insect hosts are not dependent on *Wolbachia *for heme biosynthesis, the microbe may supplement host stores or play an additional role in iron homeostasis.

In addition to the provision of metabolic cofactors, invertebrate hosts may also benefit from an additional source of nucleotides provided by *Wolbachia*. Multiple genes in this functional category (seven in *w*Bm and five in *w*Mel, Fig. 1) were affected by positive selection (see Additional file 1). Other endosymbionts, including the parasitic *Rickettsia *or beneficial *Buchnera*, scavenge nucleotides from the host environment via ATP/ADP translocases. *Wolbachia*, however encodes complete purine and pyrimidine biosynthetic pathways, and lacks the nucleotide translocase found in the closely related *Rickettsia *[10,11]. The provision of nucleotides by *w*Bm and *w*Mel could benefit their hosts during periods of rapid DNA replication and cellular division, such as during oogenesis and embryogenesis [10]. Lastly, there is widespread evidence of diversifying selection in both genomes on amino acid biosynthetic pathway genes (Fig 1 and see Additional file 1). *Wolbachia *lack many genes in the biosynthetic pathways for amino acids and therefore it is less likely they are provisioning hosts in this regard [10,11].

### Coordination of cell division with the host

The coordination of symbiont replication with host cell division is required to prevent either loss of the symbiont within the host or over replication leading to pathology within the host [1], such as that occurring with *w*MelPop. The mechanisms underlying this balancing act in *Wolbachia*-host associations are unknown. Filarial *Wolbachia *densities increase when the infection passes from the insect vector into the mammalian host [39,40]. Arthropod *Wolbachia *are also present at different densities depending on host species [20], host developmental phase [41], and tissue distribution [15,42]. For a number of insect species, *Wolbachia *has the additional challenge of dealing with host diapause where the microbe's replication would have to be slowed or stopped temporarily to maintain synchrony with host cell division [43].

**Figure 3:**
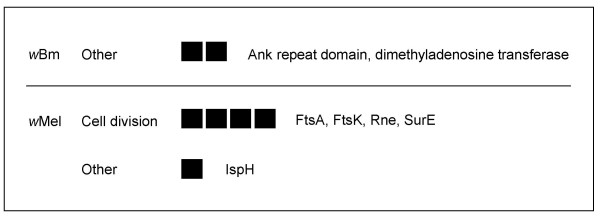
**Cellular processes**. Specific genes and d_N_/d_S _exhibiting selection (BH p = 0.001 model and Fisher's exact test) by select functional role for each genome. Each box corresponds to a significant gene in the sub role and degree of shading indicates magnitude of d_N_/d_S_. "Unrep" indicates the ratio was at the reportable limit of codeml (see methods). Names of gene products are listed in order to the right of boxes. See Additional file 1 for individual *p *values, gene ids/descriptions, WD#s, and d_N _& d_S _values.

Several genes associated with cell division particularly with regulation of growth rates, appear to be positively selected in *w*Mel (Fig. 3), including the cell division genes *ftsA *[44], *ftsK *[45], and *rne *[46]. Also affected in *w*Mel, is *surE *[47] whose expression is associated with adaptation to stressful conditions and survival of stationary phase in *E. coli*. Another rate limiting step in terms of growth and cell division that may be targeted by selection is protein synthesis. The processes of synthesis and modification of ribosomal proteins, translation factors, and base modification were heavily affected in both genomes (included in Protein synthesis, Fig. 1 and see Additional file 1). These molecular adaptations may affect rates of cell cycling indirectly by regulating rates of protein synthesis.

**Figure 4:**
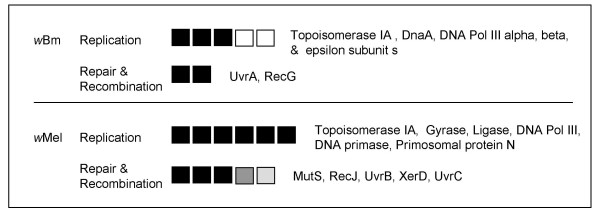
**DNA metabolism**. Specific genes and d_N_/d_S _exhibiting selection (BH p = 0.001 model and Fisher's exact test) by select functional role for each genome. Each box corresponds to a significant gene in the sub role and degree of shading indicates magnitude of d_N_/d_S_. "Unrep" indicates the ratio was at the reportable limit of codeml (see methods). Names of gene products are listed in order to the right of boxes. See Additional file 1 for individual *p *values, gene ids/descriptions, WD#s, and d_N _& d_S _values.

In both genomes, diversifying selection on genes involved with DNA replication was surprisingly common given the fundamental conserved nature of the DNA replication process (Fig. 4). A recent screen of uropathogenic *E. coli *relative to non-pathogenic strains, also revealed diversifying selection on cell division & DNA metabolism genes [26]. These pathogenic strains in their shift from commensal origins have gained the ability to invade and live inside host cells. Heightened evolutionary change in cell division and DNA replication genes may affect efficiency of growth and underpin coordination with host cell activities. This is a particular challenge for *w*Mel given the need to a adapt to a broader range of host cell types, host cell division rates, extracellular/intracellular environments, and ambient temperatures.

### Coping with Muller's ratchet

The accumulation of mildly deleterious mutations in symbionts due to repeated bottlenecking during transmission between hosts has been used to predict the irreversible degradation of symbiont genomes via the process of Muller's ratchet [48]. Selection for more effective repair or recombination systems may mitigate the effects of the ratchet upstream in the process. Both *Wolbachia *genomes appear to contain a functional set of DNA repair enzymes. Two genes in *w*Bm and five genes in *w*Mel encoding recombination and/or repair proteins were affected by positive selection. Muller's ratchet could be mitigated by genetic recombination among divergent strains of *Wolbachia *that infect a single host. However, this is not likely to occur for *w*Bm where multiple divergent strains of *Wolbachia *do not coexist within a single host. Multiple genes involved with aminoacylation of tRNAs were also affected by positive selection (see Additional file 1). These proteins ensure fidelity of translation by providing error correction [49]. The prevalence of selection was roughly equal in *w*Bm and *w*Mel (six vs. nine genes, respectively) and could represent another strategy for minimizing effects of other sources of error on protein performance.

### Variable environments

**Figure 5:**
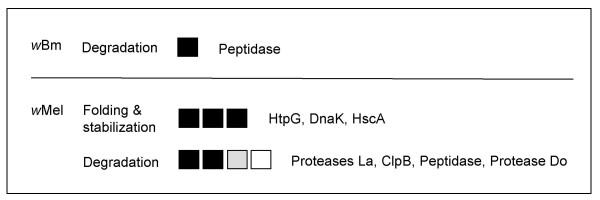
**Protein fate**. Specific genes and d_N_/d_S _exhibiting selection (BH p = 0.001 model and Fisher's exact test) by select functional role for each genome. Each box corresponds to a significant gene in the sub role and degree of shading indicates magnitude of d_N_/d_S_. "Unrep" indicates the ratio was at the reportable limit of codeml (see methods). Names of gene products are listed in order to the right of boxes. See Additional file 1 for individual *p *values, gene ids/descriptions, WD#s, and d_N _& d_S _values.

The accumulation of slightly deleterious mutants in *Buchnera *[48] by the process of Muller's ratchet has predicted the importance of chaperones in maintaining the integrity of proteins in symbionts. Evidence for positive selection on *groEL *in *Buchnera *has been interpreted as support for the action of the ratchet [50]. The protein, GroEL compensates for mildly deleterious mutations by permitting proper structures to form. This screen has identified selection on other heat shock genes and regulators of the heat shock process in including *dnaK *[50], *htpG *[51]*hscA *[52], several *clp *genes [53], and multiple proteases (Fig. 5). The prevalence of selection on genes encoding heat shock proteins is higher in *w*Mel than in *w*Bm. Several heat shock encoding genes do display evidence of diversifying in *w*Bm under less stringent rejection criteria (see Additional file 1). It is possible that the heat shock system forms part of a strategy for dealing with variable environments that may include, exposure to changing temperatures, different intracellular and extracellular environments. While the superhosts of filarial *Wolbachia *include the insect vector, the vast proportion of the nematode lifecycle is spent within a mammalian host. Shifts between mammalian and insect hosts would expose the *Wolbachia *to different temperatures and though required for long-term survival would be temporally infrequent. *Wolbachia *that infect arthropods on the other hand may be exposed to more fluctuating temperature regimes on shorter time scales as insects cannot thermoregulate and thus body temperature is more likely to vary over a 24-hour period. These *Wolbachia *are also likely to be exposed to both extracellular as well as diverse intracellular environments [15,16].

In *w*Mel one of the genes encoding part of the two-component system also exhibited evidence of positive selection (see Additional file 1, signal transduction). The two-component system forms the basis of a small-molecule signaling pathway and is thought to play a role in quorum sensing [54]. In other bacteria these pathways affect exopolysaccharide synthesis, biofilm formation, motility, cell differentiation, and virulence. Genes comprising quorum-sensing systems have previously been shown to be targets of selection [55]. Selection on this pathway in *w*Mel may indicate a mechanism for rapidly inducing widespread transcriptional changes in response to shifting habitats.

### Direct contact with the host

**Figure 6:**
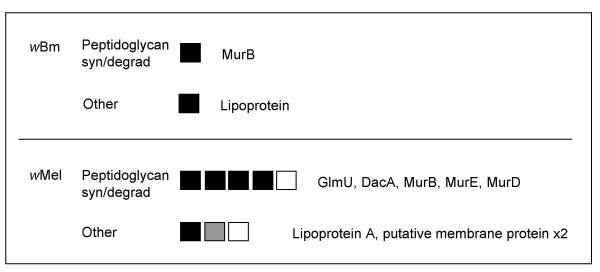
**Cell envelope**. Specific genes and d_N_/d_S _exhibiting selection (BH p = 0.001 model and Fisher's exact test) by select functional role for each genome. Each box corresponds to a significant gene in the sub role and degree of shading indicates magnitude of d_N_/d_S_. "Unrep" indicates the ratio was at the reportable limit of codeml (see methods). Names of gene products are listed in order to the right of boxes. See Additional file 1 for individual *p *values, gene ids/descriptions, WD#s, and d_N _& d_S _values.

The cell envelope and surface proteins represent the most obvious candidates for host interaction. Strong diversifying selection on genes encoding surface proteins in parasites, including *Wolbachia *[27] has been well documented [56]. Five genes in *w*Mel and one gene in *w*Bm involved with biosynthesis of peptidoglycan or cell envelope assembly were positively selected (Fig. 6). Peptidoglycan serves as one of the primary recognition molecules for the insect innate immune response and host immune systems exploit variation in the structure and metabolism of peptidoglycan for the recognition of invading bacteria [57]. It is possible that the diversifying selection in cell envelope synthesis genes has been driven by immune evasion pressure. This may be especially important in *w*Mel in times of extracellularity such as infection of hemolymph [15,16]. An alternate explanation is that because production of peptidoglycan is tightly linked with DNA replication, cell growth, and cell division, selection pressure on aspects of bacterial growth including growth phase and growth rate may have driven selection in peptidoglycan synthesis [58]. Several other genes encoding outer membrane proteins also exhibited evidence of selection in both genomes (Fig. 6 other and see Additional file 1, Hypotheticals).

### Communication with the host & extracellular environment

**Figure 7:**
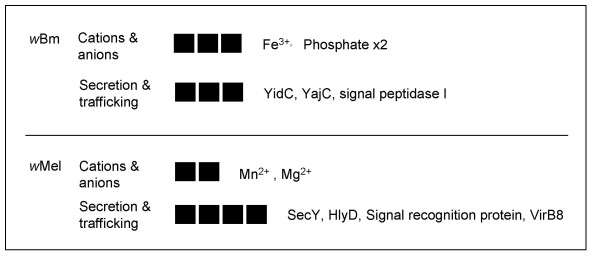
**Transport**. Specific genes and d_N_/d_S _exhibiting selection (BH p = 0.001 model and Fisher's exact test) by select functional role for each genome. Each box corresponds to a significant gene in the sub role and degree of shading indicates magnitude of d_N_/d_S_. "Unrep" indicates the ratio was at the reportable limit of codeml (see methods). Names of gene products are listed in order to the right of boxes. See Additional file 1 for individual *p *values, gene ids/descriptions, WD#s, and d_N _& d_S _values.

For an intracellular microbe, secretion and import represent the main route of communication with the host and the extracellular environment. While both *w*Mel and *w*Bm must communicate with their primary hosts, filarial *Wolbachia *may also play a role in communication with the mammalian or insect super hosts via their occupation of the hypodermal cells. These cells form channels and are involved with secretion between nematode and super hosts [59,60]. A large number of genes encoding proteins underlying secretion pathways were under selection, three in *w*Bm and four in *w*Mel (Fig. 7). These genes represent the Type I secretion system (ABC transporter), Type IV secretion system (*vir *genes), and SRP (signal recognition protein) and Sec pathways (*secY*, *yidC*, and *yajC*). The Type I system is widespread in bacteria and aids in the secretion of diverse proteins [61]. Type IV secretion facilitates host-endosymbiont communication in a broad range of intracellular bacteria [62]. The Sec pathway comprises chaperones, transport machinery, and a system of pores that carry proteins from the ribosome to the extracellular space. The SRP pathway targets proteins from the ribosome to Sec pathway pores [63].

Ankyrin repeat domain-containing proteins are common in eukaryotes and viruses and are thought to mediate protein-protein interactions. ANK encoding genes are unusually common in the *Wolbachia *genome relative to other bacteria. The ANK containing proteins are especially interesting in the *Wolbachia *system given their possible involvement in determining reproductive phenotypes or host specificity [64,65]. In *Anaplasma phagocytophilum *[66], one of these proteins is secreted into the host cell where it binds host chromatin and may affect host gene expression. Only one gene encoding an ANK protein exhibited diversifying selection in our screen (Fig. 3). The functional role of this protein in *Wolbachia *is not known.

## Conclusion

There are a number of caveats associated with the interpretation of genome wide screens for selection [67]. The methods employed here should be fairly conservative given, the use of per gene measures of d_N_/d_S _that are more likely to detect only dominant features of a gene, the statistical tests of difference between d_N _& d_S_, and use of multiple test correction procedures. We cannot completely exclude issues of saturation and increased fixation of nonsynonymous mutations in populations with small N_e _[33]. The results are also highly defined by the choice of outgroup. As more genome sequences become available future screens between strains within the *Wolbachia *genus may provide finer scale comparison among lineages. The trends identified here in terms of biological process, while not proof of adaptation, highlight the most likely points of interaction between hosts and symbionts. These areas may be targeted for empirical study in hopes of better understanding the mechanistic basis of *Wolbachia *symbiosis.

From this screen we would suggest the following hypotheses for further empirical testing. Both *w*Mel and *w*Bm may provision hosts with the following compounds or their intermediates; heme, riboflavin, ubiquinone, folate, and nucleotides. Rearing the hosts under restricted diets or more natural field conditions could reveal yet undescribed *Wolbachia *associated fitness benefits, particularly in insects. Regulating DNA replication and cell division may not only be a requirement for successful intracellularity, but also the key to adaptation to diverse cellular environments, temperatures, and host ranges in insect hosts. Enhanced DNA repair, improved translation fidelity, and the heat shock response may be adaptive responses to the action of Muller's ratchet in these small bottlenecked populations. The heat shock response in combination and two-component switching may be employed by insect *Wolbachia *to cope with variable host environments. Communication with the host is fundamental for both *Wolbachia *and evidence of diversifying selection is present in multiple secretion pathways. Insect *Wolbachia *are uniquely experiencing selection on cell envelope synthesis genes. This may reflect a greater exposure to effector molecules of the host immune system.

## Methods

### Selection and alignment of gene orthologs

*Anaplasma marginale *(St. marie's strain) [68] was selected as the outgroup as it is the closest known relative to *Wolbachia *[23]. A member of the α-proteobacteria, *A. marginale *is a pathogen of cattle that is vectored primarily by ticks [68]. Sequences of *w*Mel, *w*Bm and *A*. *marginale *protein encoding genes – 1195, 805 and 949 respectively – were obtained from the RefSeq database. Proteins were considered orthologous if each combination of Blast searches (six in this three-way comparison) identified the same gene as the best scoring match [25,69]. Ambiguous matches with little sequence similarity and very short alignments were eliminated by accepting only Blast hits with e-values less than or equal to 1 × 10^-6^. All known pseudogenes and phage sequences were excluded. The amino acid sequences for the 591 orthologs selected by the above procedure were then aligned with ClustalW ver. 1.83 [70] using default parameters and the resulting alignments back-translated into their DNA sequence, preserving patterns of indels from protein alignments.

### Inference of positive selection

The likelihood ratio test of the null hypothesis of constant rates of nonsynonymous substitutions per nonsynonymous site over synonymous substitutions per synonymous site (*d*_*N*_/*d*_*S*_) among all three lineages was performed on each triplet of genes using codon-based maximum likelihood models. The models were implemented using codeml – a program for codon-based substitution models from PAML package ver. 3.14 [24]. All models were implemented to utilize one *d*_*N*_/*d*_*S *_ratio among all amino acid sites [71]. The likelihood test was performed as a one-sided *chi-square *test of the null hypothesis H_0 _assuming one *d*_*N*_/*d*_*S *_ratio among all three lineages versus alternative hypotheses H_A _and H_B _allowing for two *d*_*N*_/*d*_*S *_ratios – one for *w*Bm or *w*Mel respectively, and a second for the remaining two lineages (branch-specific model).

Obtained log likelihood ratios were tested for significance using the upper critical value of *chi-square *distribution for one degree of freedom. The null hypothesis of constant *d*_*N*_/*d*_*S *_ratio among all three lineages was rejected when two times the log likelihood was greater than 3.84. A Benjamini & Hochberg multiple test correction [72] was employed in combination with a critical rejection value, α = 0.001. As random numbers are used to start the maximum likelihood iterations, we repeated the above analysis five times to check for convergence of the models. Average value and standard deviation of the focal lineages *d*_*N*_/*d*_*S *_ratios were used to assess model convergence. The supplemental tables report mean *d*_*N *_and *d*_*S *_values across the five replicate analyses. A number of genes with very small mean *d*_*S *_produced artificially inflated ratios at the reportable limit of codeml (999). In these cases the ratios themselves are not particularly informative (Fig. 2, *d*_*N*_/*d*_*S *_*= 999, unreportable)*. We therefore have used Fisher's exact test [73] (*p *≤ 0.001 & Benjamini & Hochberg multiple test correction) [72] for all loci to identify genes where *d*_*N *_was significantly different (and larger) from *d*_*S*_. All genes of interest reported here have therefore met both the significance criteria under the appropriate model of selection and possess report a mean *d*_*N *_that is significantly different and greater from the mean *d*_*S*_.

### Genome characteristics and assumptions of Codeml

The assumptions of codeml include similarity of base composition and codon usage patterns as well as calculable genetic distances across the sequences being compared. The *w*Bm and *w*Mel genomes have very similar base compositions, 34.1 [10] and 35.2% GC [11]. *Anaplasma marginale *is 48.9% GC [68]. A comparison of codon usage patterns between the three genomes by paired t-tests revealed no statistical differences (data not shown). Mean d_N _and d_S _values were 0.056 ± 0.002 and 0.14 ± 0.008 for *w*Bm/H_A _and 0.049 ± 0.001 and 0.10 ± 0.007 for *w*Mel/H_B_, respectively. Genetic distances are large enough that *d*_*N*_*/d*_*S *_[74] should not suffer from a time lag. Alternatively, genes experiencing a high degree of divergence and more specifically saturation could lead to overestimates of *d*_*N*_/*d*_*S*_. Anismova et al modeled the effects of various parameters including divergence on both power and accuracy of the likelihood ratio test [75]. Our datasets (three taxa, mean gene length in codons ≈ 343 Transition/transversion ratio ≈ 4.0, and median *d*_*N*_*/d*_*S *_≈ 0.3 for both H_A _and H_B_) are most similar to the reported results of experiment C. These simulations identified no type I error at α = 0.01. This study relies on a more stringent α and inspection of the data indicates that most significant genes possess high *d*_*N *_values relative to *d*_*S *_(see Additional file 1) and are therefore not likely to be artifacts of saturation.

## Authors' contributions

JB and MA carried out the genome analysis, participated in the design of the study, contributed to data interpretation, and assisted with drafting the manuscript. BS assisted with data analysis & interpretation and contributed to drafts of the manuscript. EM conceived the study, participated in the design, assisted with data interpretation, and drafted the manuscript. All authors read and approved the final manuscript.

## Supplementary Material

Additional file 1 Codeml output & Fisher's exact test for all 591 orthologs under each evolutionary model. The following information is listed for all orthologs under model H_A _or H_B_; *A. marginale*, *w*Bm, and *w*Mel gene ids, *w*Mel WD locus id, uncorrected and corrected p-value for fit to model, annotated functional roles both main and sub, N (number of nonsynonymous sites), S (number of synonymous sites), d_N_, d_S_, d_N_/d_S_, Fisher's exact test uncorrected and corrected p-values. Two additional sheets list only the subset of genes demonstrating significant evidence of positive selection. Click here for file
